# The Double-Edged Sword Effects of Teacher–AI Collaboration on Work Engagement: A Self-Determination Theory Perspective

**DOI:** 10.3390/bs16071118

**Published:** 2026-07-03

**Authors:** Jingsong Sun, Yingyu Xing, Guipeng Yuan, Qihai Cai

**Affiliations:** School of Business, Macau University of Science and Technology, Macau SAR 999078, China

**Keywords:** teacher–AI collaboration, psychological availability, work alienation, digital competency, work engagement

## Abstract

The penetration of artificial intelligence (AI) is dramatically transforming higher education, yet the effects of its pervasive adoption remain inconclusive. Drawing on self-determination theory, this study examines the double-edged effects of teacher–AI collaboration on work engagement, using psychological availability and work alienation as competing psychological mechanisms. We further examine digital competency as a boundary condition. Using three-wave time-lagged survey data collected from 468 university teachers in China, this study tested the proposed moderated mediation model through confirmatory factor analysis, hierarchical regression, and bootstrapping with 5000 resamples. The results showed that teacher–AI collaboration was positively related to work engagement. Psychological availability mediated the positive pathway from teacher–AI collaboration to work engagement, whereas work alienation mediated a negative pathway. The indirect effect via psychological availability was positive and significant, whereas the indirect effect via work alienation was negative and significant. Digital competency strengthened the positive relationship between teacher–AI collaboration and psychological availability and weakened the positive relationship between teacher–AI collaboration and work alienation, thereby amplifying the beneficial pathway and buffering the detrimental pathway. The findings offer actionable insights for university administrators to navigate digital transformation in higher education.

## 1. Introduction

As digital technologies and artificial intelligence (AI) continue to reshape workplaces, collaboration between employees and AI systems has become an important part of modern organizational life. AI is no longer only used to automate simple tasks. It now supports information search, decision-making, problem solving, content generation, and knowledge work (Li et al., 2023; Raisch & Krakowski, 2021). These changes have encouraged scholars to examine how employees work with AI, how they adapt to AI-supported roles, and how AI collaboration shapes work behavior and motivation (Budhwar et al., 2022; Chang et al., 2024; Li et al., 2025; Wu et al., 2024). This change is also evident in higher education. University teachers are knowledge workers in educational organizations. Their work involves teaching, research, assessment, student support, and academic service. Generative AI can support many of these tasks by helping teachers prepare materials, design activities, generate feedback, and improve teaching plans. Recent studies show that AI is increasingly embedded in higher education practices, including writing support, formative assessment, personalized tutoring, classroom interaction, and resource generation (Ansari et al., 2024; Memon & Kwan, 2025; Song et al., 2025; Tan, 2025). These changes suggest that AI is not only changing teaching tools but also changing teachers’ work processes and professional roles.

In this study, teacher–AI collaboration is not viewed as simple AI use. It refers to the degree to which AI is integrated into teachers’ work processes, such as problem-solving, decision-making, prediction, information evaluation, and risk identification (Kong et al., 2023; Ding et al., 2025). Prior studies suggest that this collaboration may bring clear benefits. AI can help teachers manage information, reduce repetitive tasks, and handle complex work demands (Huang & Rust, 2018; Raisch & Krakowski, 2021). It may also increase teachers’ confidence in using technology and strengthen teaching engagement (Ding et al., 2025). From a broader organizational perspective, employee–AI collaboration may also shape work behavior, work relationships, and adaptation to digital intelligence transformation (Li et al., 2025; Wu et al., 2025). However, AI collaboration may also create new tensions. AI-based work can change task boundaries, reduce employees’ sense of control, and raise concerns about skill replacement (Raisch & Krakowski, 2021; Wu et al., 2024). Recent research further shows that employee–generative AI collaboration may increase work alienation when employees feel that they lose control over work processes or become distant from the meaning of their work (Hai et al., 2025). For university teachers, these risks may be especially important. Teaching involves not only technical tasks, but also professional judgment, care, values, and human interaction. When teachers rely too much on AI, they may feel that their professional role is weakened and that they are less connected to the meaning of teaching. Although prior studies have examined AI use and AI collaboration, the double-edged effect of teacher–AI collaboration remains unclear. Many studies focus on technology acceptance, perceived usefulness, AI literacy, or AI competence. These studies explain why teachers accept and use AI, yet they offer less insight into why the same collaboration may lead to different psychological outcomes. Some teachers may gain energy and motivation from AI, while others may feel passive, controlled, or detached from their work. In this study, the term “double-edged sword effects” refers to this coexistence of beneficial and detrimental consequences. Teacher–AI collaboration may function as a resource that enhances teachers’ psychological availability and work engagement, while also creating a risk of work alienation when AI use weakens teachers’ autonomy, professional judgment, or connection to the meaning of teaching. This gap is important because work engagement reflects the energy, attention, and emotional investment that employees bring to their work (Schaufeli et al., 2002; Rich et al., 2010).

To address this gap, this study employs self-determination theory as its primary theoretical lens. Self-determination theory argues that people need autonomy, competence, and relatedness to develop high-quality motivation (Ryan & Deci, 2000). Teacher–AI collaboration may support these needs by helping teachers make better choices, handle tasks more effectively, and improve interactions with students. In this case, teachers may feel more psychologically available for work. Psychological availability refers to employees’ perception that they have sufficient physical, cognitive, and emotional resources to perform their work roles (Kahn, 1990; May et al., 2004), which may increase work engagement. At the same time, teacher–AI collaboration may frustrate teachers in terms of their basic psychological needs. If teachers feel that AI controls their work process, replaces their professional judgment, or weakens human contact, they may feel less autonomous, less competent, and less connected to the value of teaching. These experiences may lead to work alienation. Work alienation refers to a state in which employees feel powerless, meaningless, or separated from their work (Mottaz, 1981; Nair & Vohra, 2010). In this case, AI collaboration may reduce teachers’ willingness to invest energy, attention, and emotion in teaching.

Based on this logic, this study proposes a dual-pathway model. The first path is a gain path, in which teacher–AI collaboration increases psychological availability, which in turn improves work engagement. The second path is a loss path, in which teacher–AI collaboration increases work alienation and then reduces work engagement. This study also examines digital competency as a key boundary condition. Digital competency refers to teachers’ ability to understand, use, evaluate, and manage digital technologies in work settings (Claro et al., 2024; Schwarz et al., 2024). Teachers with high digital competency may use AI more actively and reflectively. They may be better able to check AI outputs, avoid overreliance, and keep control over teaching decisions. Thus, digital competency may strengthen the gain path and weaken the loss path.

This study aims to make three contributions. First, it extends employee–AI collaboration research to the higher education context by treating university teachers’ use of AI systems in daily work processes. Second, it explains the double-edged effect of teacher–AI collaboration through two opposite psychological paths. This moves beyond a single positive view of AI use. Third, it identifies digital competency as an important boundary condition that shapes the effects of AI collaboration. In this way, this study offers a motivational explanation of when and why teacher–AI collaboration promotes or harms work engagement.

## 2. Literature Review and Hypothesis Materials

### 2.1. Teacher–AI Collaboration and Work Engagement

Teacher–AI collaboration can be understood as a specific form of employee–AI collaboration in higher education. It describes the extent to which AI is embedded in teachers’ professional tasks and supports their teaching-related decisions, content creation, feedback, and student support (Kong et al., 2023; Ding et al., 2025). This concept goes beyond simple AI use. Teacher–AI collaboration focuses on the extent to which AI is embedded in meaningful teaching tasks. This view is consistent with the broader shift from automation to augmentation in organizational research. AI-based systems are increasingly viewed as tools that augment human judgment, learning, and decision-making (Wilson & Daugherty, 2018; Jarrahi, 2018; Raisch & Krakowski, 2021). In higher education, this collaborative role can appear in lesson preparation, teaching material design, feedback generation, learning assessment, and student guidance. When AI is used for these tasks, it may not only improve teaching efficiency but also change teachers’ work experience.

Work engagement refers to a positive work-related state marked by vigor, dedication, and absorption (Schaufeli et al., 2002). It also reflects the extent to which employees invest physical, cognitive, and emotional energy in their work roles (Rich et al., 2010; Guo et al., 2025). For university teachers, engagement is especially important because teaching requires more than simply completing tasks. It requires sustained attention, professional judgment, creativity, and care for students. Teachers with high engagement are more likely to invest effort in teaching design, respond to students’ needs, and improve their teaching practices. Teacher–AI collaboration may enhance work engagement by enriching teachers’ work resources. Generative AI can help teachers search for information, organize teaching materials, generate first drafts, compare teaching ideas, and prepare feedback. These functions may reduce the burden of repeated work and free teachers’ cognitive resources for high-value tasks. More importantly, AI may help teachers focus on activities that require human strengths, such as explaining complex ideas, guiding student thinking, and making value-based teaching decisions. In this sense, AI collaboration may support teachers’ work rather than simply replace it.

Recent empirical studies provide support for this view. Ding et al. (2025) found that teacher–AI collaboration significantly and positively predicts teaching engagement, and that technological self-efficacy mediates this relationship. Their findings suggest that AI collaboration can become a situational resource that changes teachers’ effort allocation, emotional investment, and classroom behavior. Similar evidence has also appeared in organizational research. Sun et al. (2026) found that employee–AI collaboration enhances work engagement among knowledge workers through meaningful work and creative self-efficacy. These studies suggest that AI collaboration may promote engagement not only by improving efficiency, but also by strengthening employees’ psychological resources and work meaning. In the context of higher education, this positive effect may be especially relevant. Teachers often need to balance teaching, research, assessment, and student support. When AI helps them manage these demands, teachers may feel that their work becomes more manageable and more responsive to students’ needs. AI can also provide new ideas for teaching design and personalized feedback, which may increase teachers’ sense of creativity and professional growth. Research on teachers’ AI-related knowledge also suggests that effective AI integration depends on the combination of technological, pedagogical, and ethical judgment rather than technical knowledge alone (Celik, 2023). Thus, when teacher–AI collaboration supports teachers’ professional agency and teaching goals, it is likely to strengthen their engagement in work. We propose that:

**Hypothesis** **1.** *The extent of teacher–AI collaboration is positively related to teachers’ work engagement.*

### 2.2. Mediating Role of Psychological Availability

Psychological availability refers to the extent to which individuals feel they have sufficient physical, cognitive, and emotional resources to perform their work roles (Kahn, 1990; May et al., 2004). It is a key psychological condition for work engagement. Even when employees find their work meaningful and feel safe at work, they may still struggle to engage if they lack energy, attention, or emotional resources. Psychological availability, therefore, captures employees’ perceived readiness and capacity to invest themselves in work. For university teachers, this condition is especially important because teaching requires sustained attention, emotional stability, and cognitive effort.

Teacher–AI collaboration may increase psychological availability by changing how teachers allocate their work resources. Generative AI can help teachers search for information, organize teaching materials, generate initial drafts, and prepare feedback. These functions may reduce the time and mental effort spent on repeated or fragmented tasks. When routine demands are partly supported by AI, teachers may have more cognitive space for tasks that require professional judgment, such as explaining concepts, guiding students, and improving course design. As a result, teachers may feel that they have more energy, attention, and emotional capacity available for teaching.

Self-determination theory provides a useful explanation for this process. The theory argues that autonomy, competence, and relatedness are basic psychological needs that support high-quality motivation (Ryan & Deci, 2000; Gagné & Deci, 2005). Teacher–AI collaboration may address these needs by expanding teachers’ choices, improving task handling, and supporting communication with students. It may support autonomy by giving teachers more choices in designing teaching materials and solving work problems. It may support competence by helping teachers handle complex tasks more effectively. It may also support relatedness when teachers use AI-assisted insights to improve communication with students and provide more personalized support. When these needs are supported, teachers are more likely to feel psychologically available for their work.

Psychological availability may further promote work engagement. Teachers with high psychological availability are more likely to bring attention, energy, and emotion into their work roles. They may feel less drained by routine demands and more willing to invest effort in teaching design, classroom interaction, and student feedback. Prior studies have shown that psychological availability is strongly linked to engagement, as it reflects whether employees have sufficient internal resources to express themselves at work (Kahn, 1990; May et al., 2004; Rich et al., 2010). Recent studies also suggest that psychological conditions remain important for engagement in changing and uncertain work environments (Albrecht et al., 2023; Wan et al., 2024).

In the context of teacher–AI collaboration, psychological availability explains how AI-enabled support is transformed into work engagement. AI collaboration may first reduce resource loss and increase teachers’ sense of control and competence. These positive experiences can strengthen teachers’ psychological availability. When teachers feel they have enough energy, focus, and emotional resources, they are more likely to invest in their teaching. Thus, psychological availability serves as a gain-based mechanism linking teacher–AI collaboration to work engagement.

**Hypothesis** **2.** *The extent of teacher–AI collaboration has a positive indirect effect on work engagement through psychological availability.*

### 2.3. Mediating Role of Work Alienation

Work alienation refers to a psychological state in which employees feel separated from their work, their work role, or the meaning of their work. Classic alienation theory describes this state through feelings such as powerlessness, meaninglessness, isolation, and self-estrangement (Seeman, 1959). In organizational research, work alienation is often linked to a loss of control, a weak sense of meaning in work, and a distance between the employee and the work role (Mottaz, 1981; Nair & Vohra, 2010). For university teachers, work alienation may appear when teaching is experienced as a mechanical task rather than a meaningful professional activity.

Teacher–AI collaboration may increase work alienation when integrating AI into teaching shifts the balance between professional agency and technical control. Generative AI can support teaching work, but it can also encroach on tasks closely related to teachers’ professional identity, such as lesson design, assessment, feedback, and student guidance. When AI becomes deeply involved in these tasks, teachers may feel that their own judgment is less central. They may also feel that teaching decisions are increasingly shaped by technical outputs rather than by educational values and human understanding. In this situation, AI collaboration may weaken teachers’ sense of autonomy and professional ownership.

This logic is consistent with self-determination theory. The theory suggests that employees are more likely to experience high-quality motivation when their needs for autonomy, competence, and relatedness are supported (Ryan & Deci, 2000; Gagné & Deci, 2005). However, these needs may be frustrated when work technologies reduce employees’ control, diminish the perceived value of their skills, or weaken the human connection. In the context of teacher–AI collaboration, teachers may feel less autonomous if they believe AI-mediated processes constrain the pace or direction of their teaching. They may feel less competent if they believe AI is replacing their expertise. They may also feel less related to students if AI-mediated outputs reduce direct human interaction. These experiences can create a sense of distance from teaching work.

Recent research on AI and work also supports this concern. AI can both enhance and diminish meaningful work, depending on how it is designed and used (Bankins & Formosa, 2023). In addition, the automation–augmentation paradox suggests that AI may improve performance while also creating new tensions around control, dependence, and human agency (Raisch & Krakowski, 2021). Empirical evidence on generative AI further indicates that employee–generative AI collaboration may have a dark side, as it can increase work alienation when employees feel they lose control over work processes or become detached from the meaning of their work (Hai et al., 2025). For educators, these external work stressors often lead to negative work outcomes, as teaching relies heavily on professional judgment, emotional labor, and human interaction (Qin et al., 2025).

Work alienation may further reduce work engagement. Engaged employees invest energy, attention, and emotion in their work roles (Rich et al., 2010). In contrast, alienated employees are less likely to see their work as meaningful or self-expressive. They may complete tasks, but with less emotional attachment and lower willingness to invest extra effort. Prior studies have shown that work alienation is associated with lower work motivation, weaker attachment to work, and less positive work behavior (Nair & Vohra, 2010; Shantz et al., 2014). For university teachers, alienation may reduce their willingness to design more effective learning activities, respond to students’ needs, or reflect on ways to improve their teaching.

Thus, work alienation can explain the loss path through which teacher–AI collaboration weakens work engagement. When AI collaboration makes teachers feel less autonomous, less competent, or less connected to the human value of teaching, teachers may experience alienation from their work. This alienation, in turn, reduces their willingness to invest energy, attention, and emotion in teaching. Therefore, teacher–AI collaboration may have a negative indirect effect on work engagement through work alienation.

**Hypothesis** **3.** *The extent of teacher–AI collaboration has a negative indirect effect on work engagement through work alienation.*

### 2.4. Moderating Role of Digital Competency

Prior studies have demonstrated that personal abilities and self-efficacy serve as vital boundary conditions regulating employees’ reactions to workplace changes (Chang et al., 2025). In this study, digital competency, as a core personal trait, describes individuals’ ability to understand, use, evaluate, and manage digital technologies in work and learning contexts. In education, this competence is not limited to technical operation. It also includes critical judgment, ethical awareness, information evaluation, communication, content creation, and problem solving (Claro et al., 2024; Schwarz et al., 2024). For university teachers, digital competency is especially important in AI-supported teaching. Teachers need to understand what AI can and cannot do. They also need to evaluate AI-generated outputs, protect academic integrity, manage data risks, and decide how to integrate AI into teaching tasks.

Digital competency may shape how teachers experience AI collaboration. Teachers with high digital competency are more likely to use AI in an active and reflective way. They can write clearer prompts, compare AI-generated outputs, identify potential errors, and adjust AI suggestions to align with teaching goals. They are also more likely to keep professional control over the final teaching decision. In this sense, digital competency is not only a skill resource. It can also serve as a capability-based support resource to help teachers maintain agencies in AI-assisted work. This view is consistent with research on teacher digital competency and AI-related pedagogical knowledge, which argues that effective AI integration requires technological, pedagogical, and ethical judgment rather than technical skills alone (Koehler & Mishra, 2009; Celik, 2023).

Digital competency may first strengthen the positive relationship between teacher–AI collaboration and psychological availability. When teachers have high digital competency, they can use AI more effectively to reduce repetitive tasks, improve lesson design, and support student feedback. They are also more able to decide when to accept, revise, or reject AI-generated content. This may increase their sense of control and competence. As a result, AI collaboration is more likely to increase their psychological availability. In contrast, teachers with low digital competency may find AI use confusing, unstable, or time-consuming. They may spend more effort learning the system, checking errors, or worrying about misuse. In this case, AI collaboration may provide less psychological support and may not increase psychological availability to the same extent.

According to self-determination theory, digital competency may help teachers make AI collaboration an autonomy- and competence-supportive experience. It allows teachers to use AI as a professional tool rather than an external force that directs their work. Recent research also suggests that teachers’ basic psychological needs are closely related to their digital competency in AI-related teaching contexts (Zhou et al., 2024). Therefore, when digital competency is high, teacher–AI collaboration should be more likely to support psychological availability.

**Hypothesis** **4a.** *Digital competency moderates the relationship between teacher–AI collaboration and psychological availability, such that this relationship is stronger when digital competency is high.*

Digital competency may also weaken the positive relationship between teacher–AI collaboration and work alienation. As discussed above, AI collaboration may increase alienation when teachers feel that AI controls the work process, weakens their professional judgment, or reduces the human meaning of teaching. High digital competency can reduce these risks. Teachers with high digital competency can better understand the limits of AI-generated content. They can also monitor AI use, correct inaccurate outputs, and keep human judgment at the center of teaching. These abilities may reduce the feeling that teaching is being controlled by technical logic.

By contrast, teachers with low digital competency may be more vulnerable to alienation in AI-assisted work. They may rely on AI outputs without fully understanding how to evaluate them. They may also feel uncertain about whether their own expertise is still needed. Such experiences can weaken autonomy and competence and may increase a sense of distance from teaching work. Prior studies on digital competency and AI in education suggest that teachers need not only access to AI tools, but also the capacity to use them critically and ethically (Celik, 2023; Küçükuncular & Ertugan, 2025). Therefore, digital competency should reduce the risk of alienation created by teacher–AI collaboration.

**Hypothesis** **4b.** *Digital competency moderates the relationship between teacher–AI collaboration and work alienation, such that this relationship is weaker when digital competency is high.*

The above arguments also suggest two moderated mediation effects. If digital competency strengthens the effect of teacher–AI collaboration on psychological availability, then the positive indirect effect of teacher–AI collaboration on work engagement through psychological availability should be stronger for teachers with high digital competency. These teachers can better leverage AI support to build energy, focus, and emotional readiness for work. Thus, they are more likely to gain engagement benefits from AI collaboration.

**Hypothesis** **5a.** *Digital competency moderates the positive indirect effect of teacher–AI collaboration on work engagement through psychological availability, such that the indirect effect is stronger when digital competency is high.*

Similarly, if digital competency weakens the effect of teacher–AI collaboration on work alienation, then the negative indirect effect of teacher–AI collaboration on work engagement through work alienation should be weaker for teachers with high digital competency. These teachers are more able to avoid overreliance, maintain professional agency, and preserve the human meaning of teaching. As a result, they are less likely to experience the loss path from AI collaboration to lower work engagement.

**Hypothesis** **5b.** *Digital competency moderates the negative indirect effect of teacher–AI collaboration on work engagement through work alienation, such that the indirect effect is weaker when digital competency is high.*

**Figure 1:**
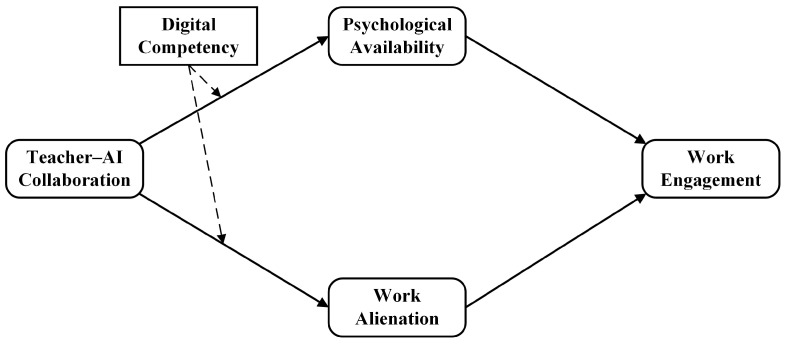
Proposed moderated mediation model.

Therefore, we propose the following moderated mediation model (See Figure 1).

## 3. Research Methods

### 3.1. Sampling and Data Collection

Data were collected from university teachers in Guangdong Province, China. A stratified sampling strategy was used, based on regional development level and university type. Five universities were selected from more developed areas and five from less developed areas. Within each university, questionnaires were distributed to teachers through online survey links.

To mitigate common method bias, this study used a three-wave, time-lagged survey design with a two-week interval between waves. At Time 1 (T1), teacher–AI collaboration, digital competency, and demographic information were measured. The demographic variables included gender, age, academic rank, teaching tenure, discipline type, and university level. About two weeks later, Time 2 (T2) measured psychological availability and work alienation. After another two-week interval, Time 3 (T3) measured work engagement.

Participants were informed that their participation was voluntary and that their responses would remain confidential. A total of 600 questionnaires were distributed. To match the three waves of data while protecting anonymity, each participant used the same self-generated code across the surveys. After removing unmatched responses and invalid questionnaires with serious missing data, extremely short completion time, or repeated response patterns, 468 valid matched responses were retained for analysis, yielding an effective response rate of 83%. The sample consisted of 220 female participants (47%) and 248 male participants (53%). Most respondents were aged 26–45, with the largest proportions in the 36–45 group (N = 140, 29.9%). As for teaching experience, most respondents had 6–10 years of experience (N = 147, 31.4%). In terms of academic rank, the majority of participants were lecturers (51.1%).

### 3.2. Measures

All measures used in this study were adapted from established scales. Because the survey was conducted in China, the original English items were translated into Chinese and then back-translated into English by two bilingual researchers. The research team compared the original and back-translated versions and revised minor wording differences to ensure semantic consistency. Some items were slightly adapted to fit the context of university teaching and the use of generative AI. Unless otherwise stated, all items were measured on a five-point Likert scale ranging from 1 = strongly disagree to 7 = strongly agree.

Teacher–AI collaboration was assessed using the five-item scale developed by Ding et al. (2025), which was adapted from the employee–AI collaboration scale proposed by Kong et al. (2023). This scale assesses the extent to which generative AI is integrated into teachers’ professional work and teaching-related tasks. A sample item is “AI participates in my decision-making process.” In this study, the wording was adapted to refer to the use of generative AI in university teaching. The Cronbach’s alpha for this scale was 0.91.

Work engagement was measured using the nine-item Utrecht Work Engagement Scale (Schaufeli et al., 2002). The scale includes three dimensions: vigor, dedication, and absorption. It has been widely used to assess employees’ positive work-related status. A sample item is “I feel full of energy when I am working.” The Cronbach’s alpha for this scale was 0.92.

Psychological availability was measured using the seven-item scale presented by Byrne et al. (2016). This scale is designed to capture individuals’ physical, cognitive and emotional readiness to engage in work, and it has been validated to have a strong correlation with work engagement in previous studies. We revised the wording of the item to fit the teaching and research contexts of university teachers. A sample item is “I am emotionally ready to deal with the demands of my work.” All items in this scale were rated on a 7-point Likert scale. The Cronbach’s alpha for this scale was 0.96.

Work alienation was assessed using the eight-item scale developed by Nair and Vohra (2010). This scale measures the extent to which employees feel detached from their work, lack meaning in their work, or experience distance from their work role. The items were adapted to the university teaching context. A sample item is “I feel less emotionally connected to my daily teaching work and more like I am simply completing tasks.” The Cronbach’s alpha for this scale was 0.93.

Digital competency was measured using a condensed nine-item scale adapted from the Generative Artificial Intelligence Competency Self-Efficacy Scale for Teachers and Researchers (GAICS-TR) developed by Xia et al. (2026). The scale reflects university teachers’ multi-dimensional self-perceived generative AI capacity, rather than only basic technical operation skills for generic digital tools. Sample items are “I can select appropriate generative AI tools to complete my teaching and research tasks” and “I can protect sensitive personal information and ensure data security when utilizing generative AI.” In the present study, digital competency was conceptualized as university faculty’s comprehensive proficiency in generative AI, encompassing ethical regulation, AI-assisted teaching, research support, and sustainable professional development. The Cronbach’s alpha for this condensed nine-item scale was 0.91.

To account for potential confounding effects, this study included gender (0 = male, 1= female), age, academic rank, and teaching tenure as control variables.

### 3.3. Data Analysis

Data analysis was conducted using SPSS 26.0 and AMOS 26.0. SPSS 26.0 was used to calculate descriptive statistics, including means, standard deviations, and Pearson correlations among the main variables. The internal consistency of each scale was examined using Cronbach’s alpha. AMOS 26.0 was then used to conduct confirmatory factor analysis and test the measurement model. Model fit was evaluated using commonly reported fit indices, including χ^2^/df, IFI, TLI, CFI, and RMSEA. Convergent validity was assessed based on factor loadings, composite reliability, and average variance extracted. Discriminant validity was examined by comparing the hypothesized five-factor model with several alternative models that combined theoretically distinct constructs.

After the measurement model was confirmed, the hypothesized structural relationships were tested. The direct relationship between teacher–AI collaboration and work engagement was examined, followed by an examination of the mediating effects of psychological availability and work alienation. Bootstrapping with 5000 resamples was used to test the significance of indirect effects; an indirect effect was considered significant if its 95% confidence interval did not include zero. To examine the moderating role of digital competency, continuous variables were mean-centered before creating interaction terms. The interaction between teacher–AI collaboration and digital competency was used to predict psychological availability and work alienation. Significant interaction effects were further interpreted using simple slope analyses at one standard deviation above and below the mean of digital competency. Conditional indirect effects were then examined to test the moderated mediation hypotheses.

## 4. Results

### 4.1. Measurement Model

We adopted the packaged item method of factor loading and conducted discriminant validity tests on five variables—namely, teacher–AI collaboration, psychological availability, work alienation, digital competency, and work engagement—through Mplus 8.0 data analysis software. The result of the five-factor model (χ^2^/df = 1.9, IFI = 0.94, TLI = 0.93, CFI = 0.94, RMSEA = 0.04) is demonstrated in Table 1. Compared with all alternative models, the five-factor model provides the best fit. Specifically, IFI, TLI, and CFI are all greater than 0.9, and RMSEA is less than 0.1, indicating that the discriminant validity of the five variables in this study is good and meets the standards.

### 4.2. Descriptive Statistics and Correlations

To evaluate the potential impact of common method variance (CMV) on our research findings, we conducted Harman’s single-factor test. All measurement items of the five core constructs were submitted to unrotated principal component analysis. As illustrated in Table 2, the first extracted component only explained 31.46% of the total variance, which is clearly below the widely accepted critical threshold of 40%. This result indicates that severe common method bias does not exist in the present dataset and will not distort subsequent hypothesis-testing outcomes. The analysis extracted five factors with eigenvalues greater than 1, which collectively accounted for 66.55% of the total variance. The emergence of multiple distinct factors further confirms that survey items belong to separate latent variables rather than converging into a single general factor driven by shared response bias.

This study conducted a correlation analysis of each variable. As shown in Table 3, teacher–AI collaboration was positively correlated with work engagement, psychological availability, digital competence, and work alienation. Psychological availability was positively correlated with work engagement, whereas work alienation was negatively correlated with work engagement. Digital competence was positively correlated with psychological availability and work engagement, and negatively correlated with work alienation. These correlation results provide preliminary support for the proposed dual-pathway model.

### 4.3. Hypothesis Testing

The hypotheses were tested using regression analysis (see Table 4) and bootstrapping procedures (see Table 5). Teacher–AI collaboration was positively associated with work engagement, supporting Hypothesis 1. This result indicates that higher levels of teacher–AI collaboration were related to higher levels of work engagement among university teachers. The mediating effects of psychological availability and work alienation were then examined. Teacher–AI collaboration was positively related to psychological availability, and psychological availability was positively related to work engagement. The bootstrapping results showed that the indirect effect of teacher–AI collaboration on work engagement through psychological availability was significant, as the 95% confidence interval did not include zero. Thus, Hypothesis 2 was supported. Teacher–AI collaboration was also positively related to work alienation, while work alienation was negatively related to work engagement. The indirect effect through work alienation was significant, supporting Hypothesis 3. These results confirm the proposed dual-pathway mechanism.

**Figure 2:**
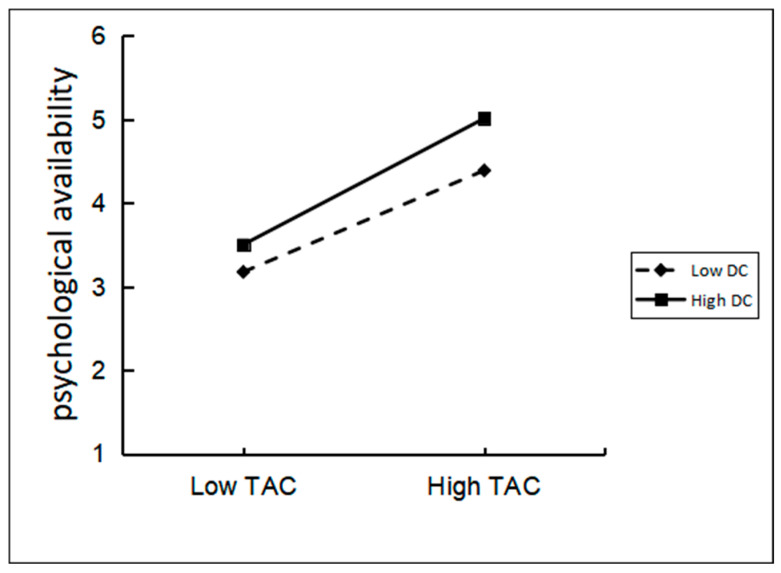
Moderating results of digital competency on the relationship between teacher–AI collaboration and psychological availability.

**Figure 3:**
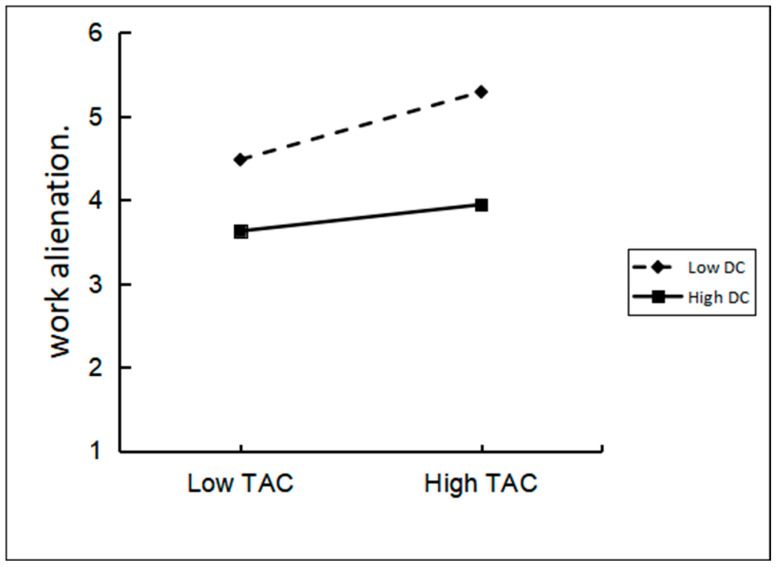
Moderating effect of digital competency on the relationship between teacher–AI collaboration and work alienation.

The moderating role of digital competence was also supported. As shown in Table 4 and Table 6, we observed a significant interaction between teacher–AI collaboration and digital competence in predicting psychological availability. Similarly, a significant interaction between teacher–AI collaboration and digital competence in predicting work alienation. Figure 2 and Figure 3 further display the interaction plot based on values plus and minus one standard deviation from the mean of digital competence. Figure 2 shows that the positive relationship between teacher–AI collaboration and psychological availability is more pronounced among individuals with high digital competence than among those with low digital competence. In contrast, Figure 3 demonstrates that the positive relationship between teacher–AI collaboration and work alienation is less prominent among individuals with high digital competence than among those with low digital competence. Consequently, these findings provide support for Hypotheses 4a and 4b.

The moderated mediation effects were examined (See Table 7). The positive indirect effect of teacher–AI collaboration on work engagement through psychological availability was stronger among teachers with high digital competence than among those with low digital competence. The negative indirect effect of teacher–AI collaboration on work engagement, mediated by work alienation, was weaker among teachers with high digital competence. The confidence intervals for both moderated mediation effects did not include zero, supporting Hypotheses 5a and 5b.

## 5. Discussion

Building on the self-determination theory, this study examined the double-edged effects of teacher–AI collaboration on university teachers’ work engagement. The results supported all hypotheses. Teacher–AI collaboration was positively related to work engagement. Psychological availability mediated the positive path from teacher–AI collaboration to work engagement, whereas work alienation mediated the negative path. Digital competence further shaped these effects. It strengthened the positive path through psychological availability and weakened the negative path through work alienation.

### 5.1. Theoretical Contributions

This study makes four main theoretical contributions. First, it extends research on employee–AI collaboration to the higher education context. Prior studies have mainly examined employee–AI collaboration in general organizational settings, focusing on career and work–family outcomes and adaptation to digital intelligence transformation (Kong et al., 2023; Li et al., 2023, 2025; Wu et al., 2025). Recent educational studies suggest that teachers are no longer only users of AI tools, but may collaborate with AI in lesson preparation, feedback generation, assessment, and student support (Kim, 2024; Ding et al., 2025; Song et al., 2025). By treating university teachers as knowledge workers in AI-supported organizations, this study shows that teacher–AI collaboration is not solely about teaching efficiency or technology adoption. It is also related to teachers’ work behavior and engagement.

Second, this study extends self-determination theory to the context of teacher–AI collaboration by showing how AI-supported teaching may reshape teachers’ professional autonomy and pedagogical judgment (Ryan & Deci, 2000; Gagné & Deci, 2005). In traditional teaching contexts, autonomy refers not only to freedom from external control, but also to teachers’ authority to define learning goals, select appropriate teaching strategies, evaluate students’ needs, and make value-based educational decisions. The findings suggest that teacher–AI collaboration may support autonomy when AI is used to reduce repetitive tasks, provide alternative instructional ideas, and expand teachers’ pedagogical options. In such cases, AI can help teachers feel more competent and psychologically available for meaningful teaching work. However, AI may also lead to frustration about autonomy when teachers feel that AI-generated outputs, algorithmic recommendations, or institutional AI systems begin to shape teaching decisions in ways that reduce their own professional control. This tension helps explain why the same AI-supported work context may generate both motivational gains and motivational losses.

Third, this study clarifies two psychological mechanisms through which teacher–AI collaboration may influence teachers’ professional identity. Psychological availability explains the gain pathway (Kahn, 1990; May et al., 2004). When AI helps teachers save time, organize information, and prepare teaching materials, teachers may have more cognitive and emotional resources to invest in activities that define their professional role, such as guiding students, designing learning experiences, and making pedagogical judgments. In this sense, AI-supported work may strengthen teachers’ identity as active educational professionals. Work alienation explains the loss pathway (Seeman, 1959; Mottaz, 1981; Nair & Vohra, 2010). When teachers experience AI as replacing their expertise, standardizing their decisions, or reducing teaching to the management of machine-generated outputs, they may feel separated from the human and value-laden meaning of education. By testing both mechanisms within the same model, this study explains why AI collaboration may energize teachers under some conditions while weakening their connection to the professional meaning of teaching under others.

Last, this study contributes to the literature on digital competence by positioning it as a capability-based boundary condition in AI-supported work. Prior studies have primarily conceptualized teachers’ digital competence as a skill structure or an ability threshold for technology integration (Celik, 2023; Claro et al., 2024). This study shows that digital competence also shapes how teachers psychologically experience AI collaboration. Teachers with higher digital competence are more able to evaluate AI outputs, avoid overreliance, and maintain control over teaching decisions. As a result, they are more likely to transform AI collaboration into psychological availability and less likely to experience work alienation. This finding supports the view that digital competence is not only a technical skill but also a capability-based resource for autonomy support.

### 5.2. Practical Implications

The findings offer three practical implications for universities seeking to improve teacher–AI collaboration. First, universities should develop AI literacy and digital competence as part of teachers’ long-term professional development. Such development should not be limited to technical training. It should include teachers’ understanding of AI limitations, ethical judgment, data privacy, output evaluation, and responsible classroom use. This approach follows the human-centered orientation of recent teacher AI competency frameworks, which emphasize that teachers need not only AI skills, but also the capacity to use AI in ways that protect human agency, ethical responsibility, and professional learning.

Second, universities should build clear human–AI work arrangements that protect teachers’ pedagogical judgment and final decision-making authority. AI can support drafting, brainstorming, organizing materials, and preparing feedback, but teachers should retain responsibility for learning goals, assessment standards, feedback quality, and value-based decisions. Universities should avoid treating AI-generated outputs as ready-made teaching decisions. Instead, AI outputs should be positioned as materials for professional review, contextual adaptation, and ethical evaluation by teachers.

Third, universities should include teachers’ professional autonomy, well-being, and identity in AI governance. The findings show that teacher–AI collaboration can enhance work engagement through psychological availability, but it can also reduce engagement through work alienation. Therefore, institutions should evaluate not only whether teachers use AI, but also whether AI use increases teachers’ sense of control, competence, teaching meaning, and professional agency. AI governance should help teachers judge when AI is useful, when AI outputs should be questioned, and when AI should not be used. Such governance is important for preventing AI integration from becoming a source of professional deskilling or identity threat.

### 5.3. Limitations and Future Directions

This study has three limitations. First, the sample was drawn from university teachers in Guangdong Province, China. Although the three-wave design strengthens the research design, regional and cultural contexts may limit the generalizability of the findings. Future studies could test the model in other provinces, countries, and higher education systems to examine whether the double-edged effect of teacher–AI collaboration remains stable across contexts.

Second, the data was collected through self-reported questionnaires. Although the time-lagged design helps reduce common method bias, self-report data may still be influenced by social desirability and subjective perceptions. Future research could combine survey data with interviews, teaching records, platform logs, peer evaluations, or student feedback. This would provide a richer understanding of how teacher–AI collaboration affects teachers’ psychological states and work behavior.

Third, this study examined teacher–AI collaboration as an overall construct. However, different AI tools and teaching tasks may lead to different outcomes. For example, the use of AI in lesson preparation, feedback generation, assessment, and student support may vary in its effects on psychological availability and work alienation. Future studies could further distinguish collaboration quality, task type, level of teacher control, and degree of AI involvement. This would help explain more precisely when AI collaboration promotes engagement and when it creates alienation.

## 6. Conclusions

By examining the double-edged effects of teacher–AI collaboration, this study shows that AI can both promote and hinder university teachers’ work engagement. Grounded in self-determination theory, the findings reveal that teacher–AI collaboration enhances work engagement through psychological availability but reduces it through work alienation. Digital competence further shapes these effects by strengthening the positive pathway and weakening the negative pathway. These findings show that teacher–AI collaboration should not be understood as uniformly beneficial or harmful. Rather, its effects depend on whether AI-supported work strengthens teachers’ psychological resources and professional agency or distances them from the autonomy, judgment, and human meaning that define teaching.

## Figures and Tables

**Table 1:** Results of confirmatory factor analyses.

Measurement Model	χ^2^	df	χ^2^/df	IFI	TLI	CFI	RMSEA
The hypothesized five-factor model	1246.94	655	1.9	0.94	0.93	0.94	0.04
Four-factor model (combining TAC and DC)	2120.08	659	3.22	0.85	0.84	0.85	0.07
Three-factor model (combining TAC, PA, and DC)	2721.48	662	4.11	0.78	0.77	0.78	0.08
Two-factor model (combining TAC, PA, WA, and DC)	4493.75	664	6.77	0.6	0.57	0.6	0.11
One-factor model (combining TAC, PA, WA, WE, and DC)	5809.8	665	8.74	0.46	0.43	0.46	0.13

Note: *N* = 468; TAC = teacher–AI collaboration; PA = psychological availability; WA = work alienation; WE = work engagement; DC = digital competency.

**Table 2:** Common method variance (CMV) results.

Component	Total	% of Variance	Cumulative %
1	11.96	31.46	31.46
2	6.35	16.71	48.17
3	4.02	10.58	58.75
4	1.92	5.05	63.79
5	1.05	2.76	66.55

**Table 3:** Descriptive statistics and correlations.

Variable	M	SD	1	2	3	4	5	6	7	8	9
1. Gender	0.47	0.50	—								
2. Age	3.71	1.47	−0.08	—							
3. Teaching year	2.82	1.55	−0.05	0.40 **	—						
4. Academic rank	2.30	0.88	−0.096 *	0.32 **	0.66 **	—					
5. Teacher–AI collaboration	4.80	1.47	0.03	−0.08	0.06	0.006	—				
6. Digital competency	5.06	1.11	−0.04	−0.02	0.08	0.02	0.51 **	—			
7. Psychological availability	3.87	1.32	0.04	−0.06	0.06	0.03	0.74 **	0.48 **	—		
8. Work alienation	4.27	0.86	−0.01	−0.06	−0.10 *	−0.10 *	0.23 **	−0.27 **	0.09	—	
9. Work engagement	3.40	0.99	−0.01	0.01	0.08	0.06	0.35 **	0.28 **	0.45 **	−0.18 **	—

Note: *N* = 468, * *p* < 0.05, ** *p* < 0.01.

**Table 4:** Regression results.

Variables	Model 1 X → Y	Model 2 X → M1	Model 3 X → M2	Model 4 X, M1, M2 → Y	Model 5 XW → M1	Model 6 XW → M2
Control variables						
Gender	−0.02	0.04	−0.04	−0.04	0.05	−0.07
Age	0.01	−0.01	0.01	0.02	−0.01	0.01
Teaching year	0.02	−0.01	−0.05	0.01	−0.02	−0.03
Academic rank	0.04	0.06	−0.05	0.01	0.06	−0.06
Independent variable						
TAC	0.24 ***	0.66 ***	0.14 ***	0.08 *	0.31 *	0.75 ***
Mediators						
PA(M1)	—	—	—	0.29 ***	—	—
WA(M2)	—	—	—	−0.27 ***	—	—
Moderator						
DC	—	—	—	—	−0.09	0.01
TAC*DC	—	—	—	—	0.06 *	−0.09 ***
R^2^	0.13	0.55	0.07	0.26	0.57	0.29
F	13.67 ***	112.91 ***	6.56 ***	23.41 ***	86.74 ***	27.04 ***

Note: *N* = 468, * *p* < 0.05, *** *p* < 0.001; TAC = teacher–AI collaboration; PA = psychological availability; WA = work alienation; WE = work engagement; DC = digital competency.

**Table 5:** Bootstrap test for the mediating effect.

	Effect	BootSE	BootLLCI	BootULCI
Total effect: TAC → WE	0.24	0.03	0.18	0.3
Direct effect: TAC → WE	0.08	0.04	0	0.16
Total indirect effect	0.16	0.03	0.09	0.22
TAC → PA → WE	0.19	0.03	0.13	0.25
TAC → WA → WE	−0.03	0.01	−0.06	−0.02

Note: *N* = 468; TAC = teacher–AI collaboration; PA = psychological availability; WA = work alienation; WE = work engagement; DC = digital competency.

**Table 6:** Bootstrap test for the moderation effect.

	DC Level	Effect	SE	LLCI	ULCI
TAC → PA	DC Low (M − 1SD)	0.54	0.04	0.47	0.62
	DC Mean	0.61	0.03	0.55	0.67
	DC High (M + 1SD)	0.68	0.05	0.58	0.77
TAC → WA	DC Low (M − 1SD)	0.38	0.03	0.31	0.44
	DC Mean	0.27	0.03	0.22	0.33
	DC High (M + 1SD)	0.17	0.04	0.09	0.24

Note: *N* = 468; TAC = teacher–AI collaboration; PA = psychological availability; WA = work alienation; WE = work engagement; DC = digital competency.

**Table 7:** Bootstrap test for moderated mediation model.

	DC level	Effect	BootSE	BootLLCI	BootULCI
TAC → PA → WE	DC Low (M−1SD)	0.16	0.03	0.11	0.21
	DC Mean	0.18	0.03	0.12	0.24
	DC High (M+1SD)	0.20	0.03	0.13	0.26
TAC → WA → WE	DC Low (M−1SD)	−0.10	0.02	−0.14	−0.07
	DC Mean	−0.07	0.01	−0.10	−0.05
	DC High (M+1SD)	−0.05	0.01	−0.07	−0.02

Note: *N* = 468; TAC = teacher–AI collaboration; PA = psychological availability; WA = work alienation; WE = work engagement; DC = digital competency.

## Data Availability

The data shown in this research are available on request from the corresponding author.
